# Low-Dose Aspirin Therapy Does not Increase the Severity of Acute Radiation Proctitis

**DOI:** 10.4021/wjon559w

**Published:** 2012-08-26

**Authors:** Hiroshi Doi, Norihiko Kamikonya, Yasuhiro Takada, Yasue Niwa, Masayuki Fujiwara, Keita Tsuboi, Hideharu Miura, Hiroyuki Inoue, Masao Tanooka, Takeshi Nakamura, Toshiyuki Shikata, Takeshi Kimura, Tohru Tsujimura, Shozo Hirota

**Affiliations:** a Department of Radiology, Hyogo College of Medicine, 1-1, Mukogawa-cho, Nishinomiya City, Hyogo, Japan; b Department of Clinical Radiology, The Hospital of Hyogo College of Medicine, 1-1, Mukogawa-cho, Nishinomiya City, Hyogo, Japan; c Department of Pharmacy, The Hospital of Hyogo College of Medicine, 1-1, Mukogawa-cho, Nishinomiya City, Hyogo, Japan; d Department of Pathology, Hyogo College of Medicine, 1-1, Mukogawa-cho, Nishinomiya City, Hyogo, Japan

**Keywords:** Radiation proctitis, Radiation colitis, Aspirin, Anticoagulant therapy, Antiplatelet therapy, Rat, Irradiation

## Abstract

**Background:**

Although anticoagulation therapy is commonly used in the prostate cancer population, there are only a few studies about the correlation between radiation proctitis and anticoagulation therapy. The purpose of the present study was to determine whether low-dose aspirin increases the severity of acute radiation proctitis in an experimental animal model.

**Methods:**

Wistar rats were used in the present study. The rats were administered either aspirin at doses of 5, 10, and 20 mg/kg, or saline, daily before and after irradiation. The rats were irradiated to the rectum as a single fraction of 25 Gy. The rectal mucosal changes of each rat were evaluated macroscopically and pathologically on the tenth day following irradiation. The findings of proctitis were graded from 0 to 4, and then were compared with regard to the status.

**Results:**

No apparent correlations were observed between the administration of aspirin and the severity of radiation proctitis in the macroscopic findings and in the morphological mucosal damage in the pathological examination. The proportion of rats with a severe degree of mucosal inflammation was 90.0%, 100.0%, 16.7% and 100.0% at 5 mg/kg, 10 mg/kg, and 20 mg/kg of aspirin, or saline, respectively. The rats receiving aspirin at the dose of 20 mg/kg showed significantly milder inflammation than the other groups (P < 0.05).

**Conclusions:**

In the present study, low-dose aspirin did not increase the severity of acute radiation proctitis. In addition, aspirin might decrease the severity of radiation-induced mucosal inflammation in the rectum.

## Introduction

Radiation proctitis is one of the major clinical complications of pelvic irradiation. The incidence of radiation proctitis depends on the radiation dose, treatment term, and fractionation schedule.

Radiotherapy is one of the standard treatment options for pelvic malignant tumors, such as uterine cervical cancer and prostate cancer. In recent years, several studies have shown better biochemical outcomes after dose-escalated radiotherapy for prostate cancer [1, 2]. Although radiotherapy is usually well tolerated, radiation proctitis is a common and potentially serious complication of radiotherapy. Acute radiation proctitis causes diarrhea, abdominal cramps, and bleeding. These effects decrease the quality of life in some patients, and often require either an interruption of therapy or other modifications that may forestall the optimal completion of the original treatment plan.

Low-dose acetylsalicylic acid (aspirin) is a well-established therapeutic regimen for the acute treatment and secondary prevention of human stroke or heart attack [3, 4]. This effect is considered to be attributable to its anti-thrombotic action, which results from the irreversible inhibition of platelet cyclooxygenase activity and thromboxane formation [5].

The cessation of low-dose aspirin or reduction of the dose of radiotherapy is often considered in patients that receive low-dose aspirin therapy because of the risk of rectal bleeding. In addition, Choe et al reported that patients taking anticoagulation therapy may have a substantial risk of bleeding toxicity from external beam radiotherapy for prostate cancer [6]. Although anticoagulation therapy is commonly used in the prostate cancer population, there is limited data about the correlation between radiation proctitis and anticoagulation therapy [6, 7].

In a previous study, we standardized an experimental rat model of acute radiation proctitis using a method to selectively irradiate the rectum without the use of surgical techniques, and reported the efficacy of Polaprezinc, an anti-ulcer drug, for the prevention of acute radiation proctitis [8].

The purpose of the present study was to determine whether low-dose aspirin increases the severity of acute radiation proctitis in an animal experimental model.

## Materials and Methods

### Animal experimental model and administration of aspirin

A total of 41 female, five weeks old, Wistar rats, weighing 90 - 120 g and were obtained from CLEA Japan Inc. (Tokyo, Japan). All of the rats were allowed to acclimate prior to the experiments. All of the animal procedures were approved by the Hyogo College of Medicine’s Institutional Animal Care and Use Committee prior to the initiation of the project.

The rats were divided into five groups as follows, aspirin 5 mg/kg/day group (ASA5; n = 10), aspirin 10 mg/kg/day group (ASA10; n = 10), aspirin 20 mg/kg/day (ASA20; n = 7), saline group (Saline; n = 7), and aspirin 20 mg/kg/day without any irradiation group (RT-/ASA20; n = 7).

All rats were administered aspirin at doses of 5, 10, or 20 mg/kg, or saline by oral gavage once per day beginning five days before irradiation. The rats were irradiated on the fifth day following the start of administration. In addition, the administration of aspirin or saline was continued daily following irradiation.

### Irradiation

A total of 34 rats, excluding the RT-/ASA20 group, were irradiated on the fifth day after the start of aspirin or saline administration. This animal model has been described previously [8, 9].

Each rat was anaesthetized with sodium pentobarbital (40 mg/kg). The rats were restrained in a vertical position and were taped by the tail to an acryl plate. Lead shielding was used to cover the rats except for a 2.5 cm long area of the lower pelvis, which contained the rectum in the middle of the field. The anus of rat was set as a superior margin of irradiation field. The rats were irradiated with a single X-ray fraction of 25 Gy at 4 MV. In addition, no filtration was used in the present study. Several rats were selected as samples to confirm the consistency between the actual dose and the delivered MU. The absorbed X-ray dose in the rectum was measured using a semiconductor detector inserted into the rectum, and the delivered MU was then revised as necessary.

### Bleeding time

The tail transection bleeding time was determined under anesthesia when each rat was irradiated. The tail was transected at 7 mm from the tip. Blood drops were removed every 15 seconds with the use of a paper filter. When blood drops were not apparent, bleeding was considered stopped. The bleeding time was determined by the seconds from tail transection to the cessation of bleeding.

## Evaluation of rectal damage

Each rat was observed daily for signs of proctitis such as diarrhea and rectal bleeding following irradiation. The clinical findings were scored as follows: 0 = no symptom; 1 = feces with mild effusion; 2 = feces with apparent effusion or soft feces; 3 = diarrhea with perianal pollution; 4 = gross bleeding.

All rats were sacrificed on the tenth day following irradiation and weighed. The distal rectum was excised for a pathological evaluation following sacrifice. The macroscopic findings were evaluated, and graded using a method described previously by Northway et al as follows; 0 = normal mucosa; 1 = edema, mild hyperemia or decreased vascularity; 2 = diffuse hyperemia, multiple punctuate areas of hemorrhage or, confluent areas of hemorrhage; 3 = presence of erosions or frank hemorrhage; 4 = ulcers [10]. In addition, the stomach was also excised and examined macroscopically in the RT-/ASA20 group.

After excision, the rectum was immediately fixed in 10% neutral buffered formalin solution following macroscopic evaluation. The rectum was sectioned and divided into 2 or 3 equal segments (from the proximal rectum to anus), and submitted for a histological analysis. All slides were stained with hematoxylin and eosin, and examined by light microscopy by a pathologist who was blinded to the groups.

The severity of proctitis in each specimen was evaluated regarding the morphological mucosal damage, the degree of inflammation, and the depth of inflammation. The morphological damage was evaluated based on the epithelial changes. In addition, the results were graded using a method that we have described previously [8]. The scores of the pathological findings are all described in Table 1.

**Table 1:** Grading of the Pathological Changes on Microscopic Examination

The Morphological Mucosal Damage
0 = Normal or minor alterations which could not be ascribed with certainly to radiation
1 = Slight crypt change without loss of epithelium
2 = Crypt change with loss of epithelium equaling less than one-half
3 = Crypt change with loss of epithelium greater than one-half
4 = Loss of epithelium through muscularis mucosa

The Degree of Inflammation
0 = Normal mucosa
1 = Minor alterations
2 = Apparent inflammation (mild inflammation)
3 = More significant inflammation (moderate inflammation)
4 = Severe inflammation.

The Depth of Inflammation
0 = No apparent inflammation
1 = Inflammatory cells extend up to edge of the mucosa (epithelium, lamina propria) but not beyond
2 = Inflammatory cells extend through the mucosa and into the submucosa
3 = Inflammatory cells infiltrate through submucosa and extends into muscularis propria
4 = Inflammatory cells extend through muscularis propria into subserosa.

The severity of proctitis was evaluated pathologically regarding the morphological mucosal damage, the degree of inflammation, and the depth of inflammation. The results were graded from 0 to 4, and then were compared.

### Statistical analysis

The results are presented as the means ± standard deviation (SD). The results of the mucosal damage were divided into a milder or more severe status, milder findings were defined as Grades 0 to 2, and severe findings included Grades 3 and 4. The results were compared as applicable. The relationship between the groups was assessed using a two-tailed Fisher’s exact test. P*-*values < 0.05 were considered to be statistically significant. The Holm method was used to adjust the P-values in multiple testing. Tukey's all-pairwise-comparison test was used to identify differences in the bleeding time among the groups. The statistical difference in the bleeding time in all groups treated with ASA and in the Saline group was determined by the Mann-Whitney U test. A difference with P < 0.05 was considered to be significant.

## Results

One rat in the ASA20 group died before irradiation. Another rat in the Saline group died on the fifth day following irradiation with no apparent symptoms of proctitis.

These two irradiated rats were excluded from the examination of the rectum.

### Body weight

The mean body weights of the groups on the day when the rats were irradiated were 128.2 ± 5.7 g, 128.0 ± 3.6 g, 125.2 ± 2.7 g, and 129.2 ± 3.0 g in the ASA5 group, the ASA10 group, the ASA20 group, and the Saline group, respectively. The homogeneity of the groups was confirmed by analyzing the bodyweights using the one-way analysis of variance (P = 0.14) and Bartlett’s test (P = 0.28).

The mean body weights of the groups on the tenth day following irradiation were 150.1 ± 8.6 g, 144.4 ± 6.7 g, 136.4 ± 7.8 g, and 143.6 ± 6.1 g in the ASA5 group, the ASA10 group, the ASA20 group, and the Saline group, respectively. The one-way analysis of variance (P = 0.013) was significant, therefore differences among the groups were determined using Tukey’s test (P < 0.01). Consequently, a significant difference was observed between the ASA5 group and the ASA20 group. However, no significant differences were found among the groups using Bartlett’s test (P = 0.81).

### Prolongation of bleeding time

Aspirin caused no apparent mucosal damage to the stomach and the rectum in the RT-/ASA20 group. Two rats in the ASA10 group were excluded from the evaluation because of a technical error.

The bleeding time was 477 ± 214, 564 ± 219, 560 ± 174, and 369 ± 116 seconds in the ASA5 group, the ASA10 group, the ASA20 group, and the Saline group, respectively. The mean bleeding time was 527 ± 202 seconds in all rats which received ASA.

The addition of aspirin tended to prolong the bleeding time at more than 10 mg/kg, although there were no significant differences among the groups. In addition, the bleeding time was significantly prolonged in all groups that received ASA in comparison to the Saline group.

### Radiation proctitis

The results of the clinical findings and the macroscopic findings are shown in Table 2.

**Table 2:** The Clinical and Macroscopic Findings

Clinical findings					
Group (number)	Grade 0	Grade 1	Grade 2	Grade 3	Grade 4
ASA5 (n = 10)	0 (0.0)	1 (10.0)	7 (70.0)	2 (20.0)	0 (0.0)
ASA10 (n = 10)	0 (0.0)	8 (80.0)	1 (10.0)	1 (10.0)	0 (0.0)
ASA20 (n = 6)	0 (0.0)	1 (16.7)	2 (33.3)	2 (33.3)	1 (16.7)
Saline (n = 6)	0 (0.0)	5 (83.3)	0 (0.0)	1 (16.7)	0 (0.0)
RT-/ASA20 (n = 7)	7 (100.0)	0 (0.0)	0 (0.0)	0 (0.0)	0 (0.0)

Comparison	P-value	Comparison	P-value
ASA5 vs. ASA10	0.61	ASA10 vs. ASA20	0.12
ASA5 vs. ASA20	0.30	ASA10 vs. Saline	1.00
ASA5 vs. Saline	1.00	ASA20 vs. Saline	0.30

Macroscopic findings					
Group (number)	Grade 0	Grade 1	Grade 2	Grade 3	Grade 4
ASA5 (n = 10)	0 (0.0)	0 (0.0)	8 (80.0)	2 (10.0)	0 (0.0)
ASA10 (n = 10)	0 (0.0)	5 (50.0)	5 (50.0)	0 (0.0)	0 (0.0)
ASA20 (n = 6)	0 (0.0)	2 (33.3)	3 (50.0)	1 (16.7)	0 (0.0)
Saline (n = 6)	0 (0.0)	2 (33.3)	3 (50.0)	1 (16.7)	0 (0.0)
RT-/ASA20 (n = 7)	2 (28.6)	2 (28.6)	3 (42.9)	0 (0.0)	0 (0.0)

Comparison	P-value	Comparison	P-value
ASA5 vs. ASA10	0.24	ASA10 vs. ASA20	0.38
ASA5 vs. ASA20	1.00	ASA10 vs. Saline	0.38
ASA5 vs. Saline	1.00	ASA20 vs. Saline	1.00

The results of the clinical and macroscopic findings are presented as numbers, with percentages in parentheses. There were no significant differences among the irradiated groups in either the clinical or the macroscopic findings.

According to the clinical findings, the proportion of severe changes was 20.0%, 10.0%, 50.0% and 16.7% in the ASA5 group, the ASA10 group, the ASA20 group, and the Saline group, respectively. Almost all irradiated rats showed some type of diarrhea, except for one rat with hematochezia in the ASA20 group.

The proportion of severe macroscopic changes in the rectum was 20.0%, 0.0%, 16.7% and 16.7% in the ASA5 group, the ASA10 group, the ASA20 group, and the Saline group, respectively. There were no significant differences among the irradiated groups in either the clinical or the macroscopic findings.

**Figure 1:**
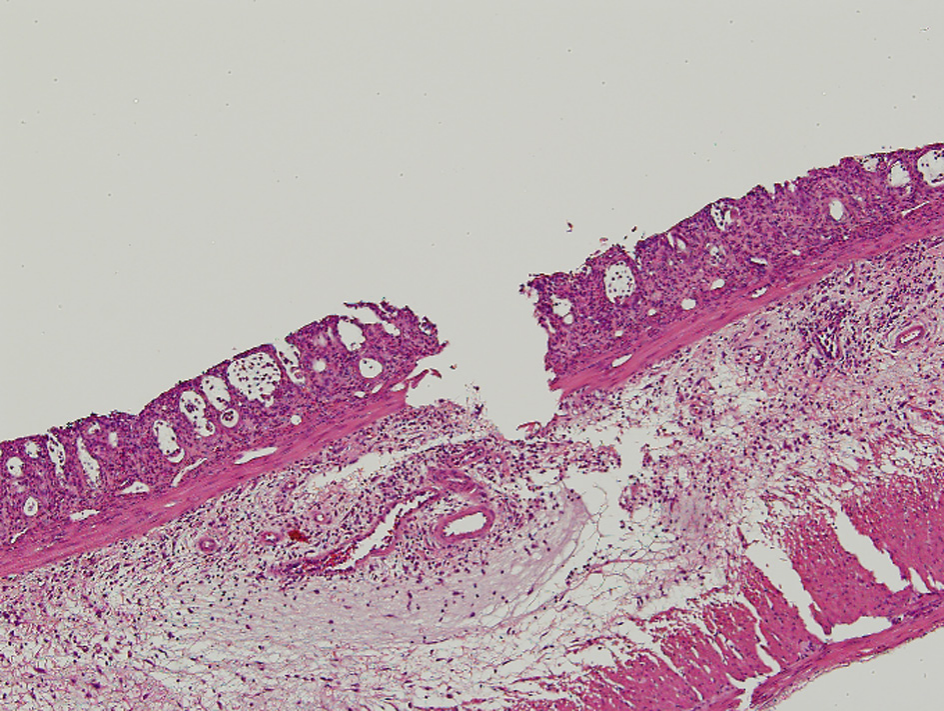
In the pathologic findings, crypt architectural distortion with inflammatory cells, loss of the columnar shape, and the submucosal edema were observed. The loss of epithelium reached submucosa through muscularis mucosa for Grade 4 in the morphological damage. The degree of inflammation was severe and corresponded to Grade 4.

Apparent mucosal changes were observed in all specimens of the irradiated rats in the pathological examination. The pathological findings are shown in Figure 1. The results of the pathological findings are all shown in Table 3. Three specimens from the ASA10 group and one specimen from the Saline group were lost in the fixation process.

**Table 3:** The Pathological Findings

Morphological mucosal damage					
Group (number)	Grade 0	Grade 1	Grade 2	Grade 3	Grade 4
ASA5 (n = 10)	0 (0.0)	0 (0.0)	3 (30.0)	2 (20.0)	5 (50.0)
ASA10 (n = 7)	0 (0.0)	0 (0.0)	2 (28.6)	3 (42.9)	2 (28.6)
ASA20 (n = 6)	0 (0.0)	0 (0.0)	3 (50.0)	0 (0.0)	3 (50.0)
Saline (n = 5)	0 (0.0)	0 (0.0)	1 (20.0)	1 (20.0)	3 (60.0)
RT-/ASA20 (n = 7)	7 (100.0)	0 (0.0)	0 (0.0)	0 (0.0)	0 (0.0)

Comparison		P-value	Comparison		P-value
ASA5 vs. ASA10		1.00	ASA10 vs. ASA20		0.59
ASA5 vs. ASA20		0.61	ASA10 vs. Saline		1.00
ASA5 vs. Saline		1.00	ASA20 vs. Saline		0.55

Degree of inflammation					
Group (number)	Grade 0	Grade 1	Grade 2	Grade 3	Grade 4
ASA5 (n = 10)	0 (0.0)	0 (0.0)	1 (10.0)	8 (80.0)	1 (10.0)
ASA10 (n = 7)	0 (0.0)	0 (0.0)	0 (0.0)	3 (42.9)	4 (57.1)
ASA20 (n = 6)	0 (0.0)	0 (0.0)	5 (83.3)	1 (16.7)	0 (0.0)
Saline (n = 5)	0 (0.0)	0 (0.0)	0 (0.0)	2 (40.0)	3 (60.0)
RT-/ASA20 (n = 7)	6 (85.7)	1 (14.3)	0 (0.0)	0 (0.0)	0 (0.0)

Comparison		P-value	Comparison		P-value
ASA5 vs. ASA10		1.00	ASA10 vs. ASA20		0.0047
ASA5 vs. ASA20		0.0076	ASA10 vs. Saline		(avaluative)
ASA5 vs. Saline		1.00	ASA20 vs. Saline		0.015

Depth of inflammation					
Group (number)	Grade 0	Grade 1	Grade 2	Grade 3	Grade 4
ASA5 (n = 10)	0 (0.0)	0 (0.0)	8 (80.0)	0 (0.0)	2 (20.0)
ASA10 (n = 7)	0 (0.0)	0 (0.0)	3 (42.9)	0 (0.0)	4 (57.1)
ASA20 (n = 6)	0 (0.0)	0 (0.0)	6 (100.0)	0 (0.0)	0 (0.0)
Saline (n = 5)	0 (0.0)	0 (0.0)	2 (40.0)	0 (0.0)	3 (60.0)
RT-/ASA20 (n = 7)	7 (100.0)	0 (0.0)	0 (0.0)	0 (0.0)	0 (0.0)

Comparison		P-value	Comparison		P-value
ASA5 vs. ASA10		0.16	ASA10 vs. ASA20		0.070
ASA5 vs. ASA20		0.50	ASA10 vs. Saline		1.00
ASA5 vs. Saline		0.25	ASA20 vs. Saline		0.061

The results of the pathological findings are presented as numbers, with percentages in parentheses. There were no significant differences among the irradiated groups in the morphological mucosal damage and in the depth of inflammation. Whereas, significant differences were observed between the ASA20 group and the other irradiated groups in the degree of inflammation.

In the microscopic examination, there were no apparent pathological changes in the rectal mucosa of the rats in the RT-/ASA20 group.

The proportion of rats with severe morphological mucosal damage was 70.0%, 71.4%, 50.0% and 80.0% in the ASA5 group, the ASA10 group, the ASA20 group, and the Saline group, respectively. There were no significant differences in the morphological findings among the irradiated groups.

Regarding the degree of inflammation, the proportion of rats with severe status was 90.0%, 100.0%, 16.7% and 100.0% in the ASA5 group, the ASA10 group, the ASA20 group, and the Saline group, respectively. There were significant differences between the ASA20 group and the other irradiated groups.

Regarding the depth of inflammation, the proportion of rats with deep inflammation was 20.0%, 57.1%, 0.0 %, and 60.0% in the ASA5 group, the ASA10 group, the ASA20 group, and the Saline group, respectively. Although no significant differences were observed in the depth of inflammation among the irradiated groups, only the rats in the ASA20 group did not show any Grade 4 findings.

## Discussion

Aspirin remains the most widely used drug for the prevention of vascular events. It has shown a positive effect in acute myocardial infarction patients and for the prevention of atherothrombotic events and strokes [3, 4]. Although aspirin is commonly used in the prostate cancer population, there have been only a few reports regarding the effect of aspirin on radiation proctitis [1]. Therefore, it remains unclear whether aspirin has a positive or negative effect on radiation proctitis.

In addition, the reports about the effects of anti-platelet agent on the prolongation of the bleeding time in rat models are limited. Therefore, it was necessary to examine the most suitable dose of aspirin in rats that could produce a prolongation of the bleeding time, and thereby indicating effective anti-platelet.

The effects of aspirin are considered to be attributable to its anti-thrombotic action, which results from the irreversible inhibition of platelet cyclo-oxygenase activity and thromboxane formation [5]. However, aspirin also inhibits the cyclo-oxygenase enzymes in the vascular endothelium, which is the source of prostacyclin, a potent inhibitor of platelet aggregation and a vasodilator [11].

No significant difference has been reported to be observed between aspirin doses of 300 to 325 mg daily and doses of 75 to 100 mg daily with respect to the same outcome in patients with acute coronary syndrome, thus indicating that even a low-dose can be effective in humans [12].

Low-dose aspirin has been described as a dose of 5 to 100 mg in rat models [13-16]. Wang et al and Ishizuka et al have reported the efficacy of low-dose aspirin for cerebral ischemic injury in a rat model [13, 14]. In these previous studies, the efficacy of aspirin tended to increase with the dose. However, Serebruany et al have reported that increased doses of aspirin tend to increase bleeding rates based on analysis of multiple clinical trials [17]. Nevertheless, the relationship between the dose of aspirin and its antiplatelet effect in rats has been unclear.

In addition, aspirin can cause gastrointestinal mucosal injuries. The mechanisms of aspirin-induced gastric mucosal injury have been reported to be due to the direct effect of aspirin on the gastric mucosa, and resulting from either a reduction in the intracellular pH or due to the dysfunction of the gastric epithelial barrier [18, 19].

Fiorucci et al have reported that doses of aspirin of 30 mg/kg produced significant gastric damage in rats in a dose-response study [20]. In addition, Sparatore et al reported that aspirin caused gastric mucosal damage at a dose of 23 mg/kg [21]. Therefore, we chose to use doses of 5 mg/kg to 20 mg/kg of aspirin for the present study to avoid the incidence of such injuries. As a result, we were able to establish an experimental animal model of radiation proctitis in rats on antiplatelet therapy without any apparent aspirin-induced mucosal damage in the rectum or stomach.

In the present study, there were no apparent correlations between the administration of aspirin and the severity of radiation proctitis in the clinical findings, in the macroscopic findings, or in the morphologic mucosal damage as observed in pathological examination, although the bleeding time was prolonged by the use of aspirin, especially at more than 10 mg/kg. These results suggest that aspirin did not increase the severity of acute radiation proctitis induced by radiotherapy. Therefore, pelvic irradiation does not seem to be an indication for the cessation of low-dose aspirin therapy or a dose reduction of radiotherapy.

Mennie et al reported that high-dose aspirin reduced the severity of abdominal symptoms in patients with uterine cancer [22]. In addition, Midaoui et al have reported that aspirin has antioxidative properties at 100 mg/kg in rats based on the determination of O^2-^ levels [16]. In the present study, The ASA20 group showed a tendency to have lower grades of inflammation than the other groups. These results might be attributable to the anti-inflammatory effects of aspirin.

In the present study, the mucosal changes were evaluated on the 10th day after irradiation with a single X-ray fraction of 25 Gy. This technique and the similar follow-up term have been applied in several reports, although a single fraction of 25 Gy is not currently clinically used [8-10, 23-25]. In addition, we have reported the application of this animal experimental model in the previous study [8]. As a result, the apparent mucosal changes were observed in all irradiated rats and were also assessed well in the present study. Moreover, acute radiation proctitis has been reported to be a strong predictor for late radiation proctitis [26, 27].

Late radiation-induced rectal injury is thought to result from tissue ischemia attributable to capillary damage and microvascular occlusion [28]. In addition, we have previously reported the correlation between the sequential changes of rectal mucosa and microangiopathy [29]. It is uncertain whether antiplatelet therapy including low-dose aspirin impacts the severity of late radiation proctitis after long-term use, although anticoagulate therapy has been reported to increase the risk of rectal bleeding after radiotherapy for prostate cancer [6]. However, no apparent impact of the administration of aspirin on the on acute radiation proctitis was observed in the present study. Acute radiation proctitis has been reported to be a predictor of late radiation proctitis [26, 27]. In addition, Konturek et al have reported that the area of gastric lesions was significantly smaller after four days of treatment with aspirin following the first exposure, and also reported that this reduction was accompanied by a significant increase in gastric blood flow [30]. Therefore, we suggest that the continuation of low-dose aspirin therapy can be tolerable for the patients received pelvic irradiation.

As a limitation of this study, the present study used only a small number of rats. Therefore, it may be necessary to confirm our results in a study of a large number of animals. The replication of these findings in a new study based on the present study, and a clinical study, are presently under investigation to confirm the influence of low-dose aspirin on radiation proctitis in more detail.

To conclude, we found that the administration of low-dose aspirin did not increase the severity of acute radiation-induced mucosal damage using an animal experimental model in the present study. Conversely, the administration of aspirin may decrease the severity of the mucosal inflammation induced by irradiation in the rectum.
